# Extranodal non-Hodgkin’s lymphoma of the gingiva in an HIV seropositive patient

**DOI:** 10.4103/2589-0557.75008

**Published:** 2010

**Authors:** Karthikeya Patil, V. G. Mahima, H. S. Srikanth

**Affiliations:** Department of Oral Medicine and Radiology, J.S.S. Dental College and Hospital, Mysore 15, India

**Keywords:** Gingival NHL, lymphoma, non-Hodgkin’s, gingival malignancy

## Abstract

Among the myriad manifestations of HIV, non-Hodgkin’s lymphomas (NHL) are considered as the second most common malignancies after Kaposi’s sarcoma. HIV-associated NHLs are extranodal and have a predilection for sites in the head and neck region in 50–60% of cases. Of all the extranodal NHLs, oral cavity constitutes only 25%. It is now considered that oral NHL serves as the first indicator of HIV infection.

## INTRODUCTION

Lymphomas are a heterogeneous group of malignancies that arise in lymphocytic progenitor cells. The association between HIV and lymphoproliferative malignancy is a well-documented phenomenon. Approximately 3% of HIV-positive patients will develop a lymphoma in the course of the disease. Non-Hodgkin’s lymphomas (NHL) are 60 times more common in HIV/AIDS than in the general population.[1,2] Oral NHLs commonly present as growth and ulceration, and it is found that the gingiva is a favored site for their occurrence.

## CASE REPORT

**Figure 1:**
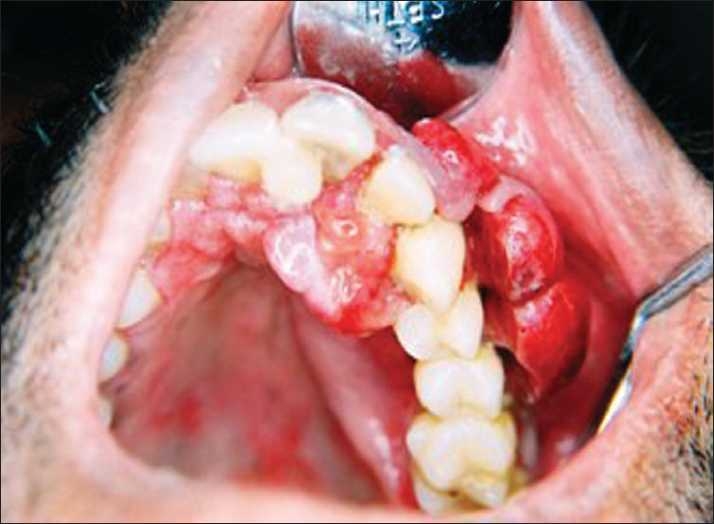
Intraoral photograph showing the multinodular swelling on the buccal gingiva and its extension onto the palatal aspect

A 34-year-old male patient consulted the Department of Oral Medicine and Radiology, with a complaint of swelling on his upper left back gums since 3 months [Figure 1]. The swelling had an insidious onset, gradually and constantly increasing in size. There was no preceding pain in the regional teeth. Occasional bleeding and mobility of the upper left back teeth was reported. Associated symptoms of loss of weight and appetite were present.

Medical history was significant in that he was diagnosed with pulmonary tuberculosis for which he was under treatment.

Personal history was significant as well in that he had visited commercial sex workers, repeatedly.

Intraorally maxillary left buccal gingiva with respect to the teeth 22, 23, 24, 25, showed a sessile swelling, measuring 6 × 3 cm in size, having a fiery red color with superficial ulcerations and a multinodular appearance. It was nontender to palpation and firm in consistency and did not induce any discharge. The swelling had also extended onto the maxillary palatal gingiva in relation to tooth 21, 22, 23. The teeth 21, 22, 23, 24, 25 exhibited grade II mobility. Periodontal pockets were not detected in the region of complaint [Figure 1].

The mid dorsum of the tongue showed an irregular area of depapillaton with diffuse erythema. The hard palate demonstrated diffuse erythema with no evidence of scrapable or nonscrapable white lesions in association, the clinical impression of which was chronic erythematous candidiasis. Rest of the oral mucosa appeared normal.

This clinical picture raised a strong suspicion of an underlying immunocompromised status for which HIV-(Western Blot) test was advised, and the patient was found to be reactive for HIV 1 and 2.

**Figure 2:**
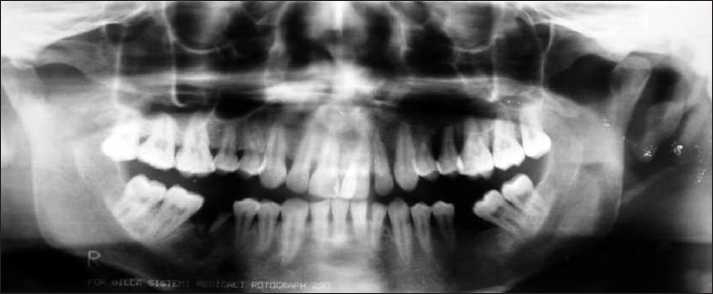
Panoramic radiograph showing a diffuse radiolucency in the maxillary left alveolar bone suggestive of loss of bone

Panoramic radiograph [Figure 2] and IOPARs taken in the region of complaint showed loss of supporting alveolar bone, with no evidence of any central pathology. A chest radiograph showed the presence of diffuse radiolucencies involving the right upper lobe of the lung. Complete hemogram revealed macrocytic anemia and raised ESR (32 mm/hr).

**Figure 3:**
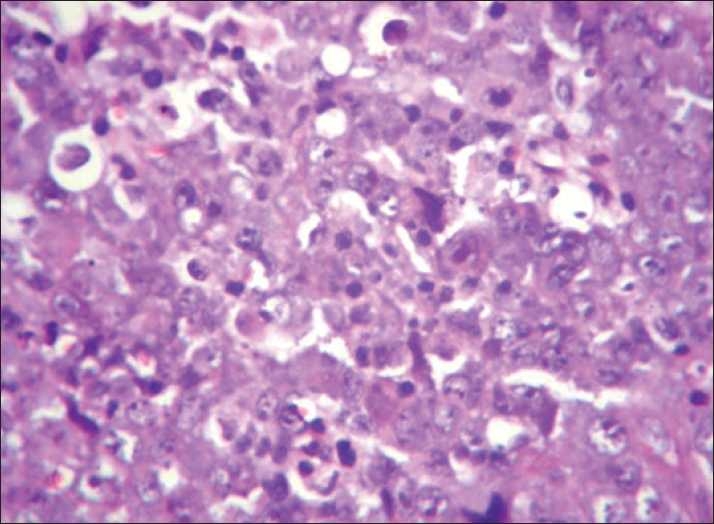
H and E under 40×

**Figure 4:**
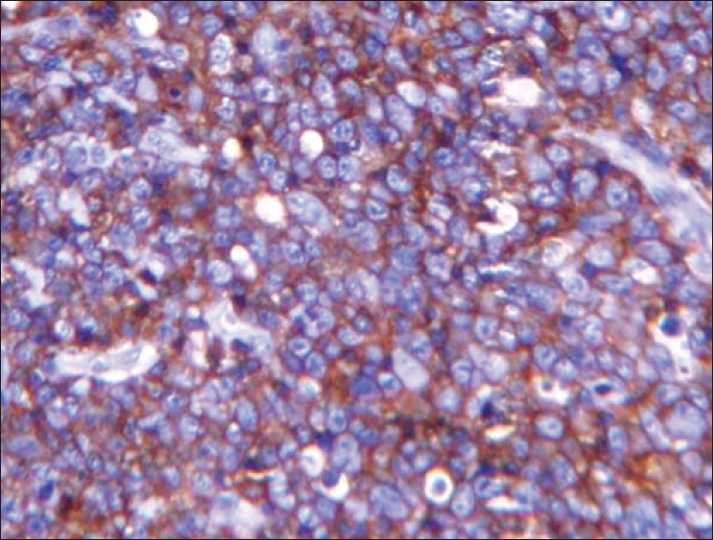
IHC – diffuse positivity of the round cells for CD 20

An incisional biopsy was performed from the gingiva. The lesion on H and E showed ulcerated stratified squamous epithelium with underlying connective tissue showing diffuse infiltration by large round cells with vesicular nuclei, single prominent eosinophilic nucleus, and scanty cytoplasm. Increased mitotic activity was evident in the cells. Areas of necrosis and pyknotic cells were also evident. The impression was that of intermediate grade NHL [Figure 3]. Immunohistochemistry was performed with CD 20 marker that showed diffuse positivity in the cells and confirmed the diagnosis of B-cell NHL [Figure 4].

The sections from the gingiva were also stained for AFB, which turned out to be negative.

Final diagnosis was that of HIV-associated NHL of maxillary gingiva.

The patient was referred to a regional oncology center for the management of NHL as well as to a physician for the treatment of TB and for ART. The patient did not report to either of the treatment centers even on repeated reminders and ultimately succumbed 4 months after the diagnosis.

## DISCUSSION

Among the vivid manifestations of HIV, NHL is considered as the second most common malignancy next to Kaposi’s sarcoma.[1]

Lymphomas, both Hodgkin’s and non-Hodgkin’s, are a heterogeneous group of malignancies that arise in lymphocytic progenitor cells. Lymphomas may arise in the lymphnodes or other extranodal organs and can metastasize to different organs.[3]

NHLs are classified depending on the cell of origin as B-cell and T-cell lymphomas, the B-cell variant being more common. The lesions are also further divided histopathologically as either follicular or diffuse type. Oral NHLs are commonly B-cell diffuse type.[4] Histologic subtypes of primary NHLs[5] have been described, which are summarized in Table 1. The immunoblastic, lymphoblastic, and small noncleaved cell type of NHLs are considered as high-grade variety with poor prognosis.[6]

**Table 1:** Histologic subtypes of primary Non-Hodgkin’s lymphomass

Subtype	Percentage of occurrence
Large B-cell	43
Mixed small and large cell	20
Immunoblastic	17
Lymphoblastic	9
Small cleaved cell	7
Small noncleaved cell	3
Small lymphocytic cell	1

HIV-associated NHLs are extranodal and have a predilection for sites in the head and neck region in 50–60% of cases. Of all the extranodal NHLs, oral cavity constitutes to only 25%, and 0.6% of them arise as a growing mass.[5] The other manifestations can be ulcerations, mobility or early loss of teeth, delayed healing of extraction sockets, masses from extraction sockets, or trigeminal neuropathy.[7] Intraorally, the most common sites are the vestibules, the buccal gingiva, and the palatal mucosa.[8] The lesions in the present case presented as nodular mass over the gingivae.

A study conducted to determine the gingival manifestations in HIV patients revealed neoplasms on the gingiva to be rare with only 6% occurrence. NHLs of gingiva have a prevalence 0.6%.[9] Sporadic case reports of NHLs involving gingiva are available, most of lesions have been in the form of growths.[1,2,5,7] A case report where five NHLs in the form of growths that occurred over the maxillary and mandibular gingivae in an HIV-seropositive patient is available in the literature.[10] Oral NHLs that appear as growths will show ulcerations covered with pseudomembranes, with pain, tooth mobility, and paraesthesia.[5] In the present case, the lesion exhibited fiery red color, covered with superficial necrosis, and was not painful but had caused mobility of teeth.

Histopathologically, the lesions exhibit connective tissue infiltrated by numerous round cells, vesicular nuclei, and prominent nucleoli with scanty cytoplasm. Atypical mitosis along with tangible body macrophages may be seen.[1] Immunohistochemistry has further contributed toward the classification of NHL. CD 20, CD 79a, MB2, CD 30 are the B-cell markers, CD 3 and CD 45RO are T-cell markers.[3]

Treatment of HIV-associated NHLs includes HAART as well as chemotherapy and radiotherapy. The standard multidrug regimen consisting of methotrexate, bleomycin, doxorubicin, cyclophosphamide, vincristine, and dexamthasone are administered.[1] A case report of spontaneous regression of an HIV-associated plasmablastic oral NHL with HAART alone is available in the literature.[11]

The median survival rate in HIV-related NHLs is found to be 5–7 months. It is suggested that patients with no extranodal disease, no prior HIV-related illness, less immunodeficiency (CD4 cell > 100 cells/mm^3^) will enjoy a better prognosis with a survival duration of 1–2 years.[11]

## CONCLUSION

Of the numerous manifestations of HIV, NHL though is the second most common malignancy; its occurrence on the gingiva is still rare. Many a times, the oral lesions indicate the underlying immunocompromised state. Swift identification and prompt therapy are the key for better patient prognosis.
